# The Mode of Action of Recombinant *Mycobacterium tuberculosis* Shikimate Kinase: Kinetics and Thermodynamics Analyses

**DOI:** 10.1371/journal.pone.0061918

**Published:** 2013-05-06

**Authors:** Leonardo Astolfi Rosado, Igor Bordin Vasconcelos, Mário Sérgio Palma, Vincent Frappier, Rafael Josef Najmanovich, Diógenes Santiago Santos, Luiz Augusto Basso

**Affiliations:** 1 Centro de Pesquisas em Biologia Molecular e Funcional (CPBMF), Instituto Nacional de Ciência e Tecnologia em Tuberculose (INCT-TB), Pontifícia Universidade Católica do Rio Grande do Sul (PUCRS), Porto Alegre, RS, Brazil; 2 Programa de Pós-Graduação em Medicina e Ciências da Saúde, PUCRS, Porto Alegre, RS, Brazil; 3 Programa de Pós-Graduação em Biologia Celular e Molecular, PUCRS, Porto Alegre, RS, Brazil; 4 Laboratório de Biologia Estrutural e Zooquímica, Centro de Estudos de Insetos Sociais, Departamento de Biologia, Instituto de Biociências de Rio Claro, Universidade Estadual Paulista (UNESP), Rio Claro, SP, Brazil; 5 Department of Biochemistry, Faculty of Medicine, Université de Sherbrooke, Sherbrooke, Québec, Canada; Oak Ridge National Laboratory, United States of America

## Abstract

Tuberculosis remains as one of the main cause of mortality worldwide due to a single infectious agent, *Mycobacterium tuberculosis*. The *aroK*-encoded *M. tuberculosis* Shikimate Kinase (*Mt*SK), shown to be essential for survival of bacilli, catalyzes the phosphoryl transfer from ATP to the carbon-3 hydroxyl group of shikimate (SKH), yielding shikimate-3-phosphate and ADP. Here we present purification to homogeneity, and oligomeric state determination of recombinant *Mt*SK. Biochemical and biophysical data suggest that the chemical reaction catalyzed by monomeric *Mt*SK follows a rapid-equilibrium random order of substrate binding, and ordered product release. Isothermal titration calorimetry (ITC) for binding of ligands to *Mt*SK provided thermodynamic signatures of non-covalent interactions to each process. A comparison of steady-state kinetics parameters and equilibrium dissociation constant value determined by ITC showed that ATP binding does not increase the affinity of *Mt*SK for SKH. We suggest that *Mt*SK would more appropriately be described as an *aroL*-encoded type II shikimate kinase. Our manuscript also gives thermodynamic description of SKH binding to *Mt*SK and data for the number of protons exchanged during this bimolecular interaction. The negative value for the change in constant pressure heat capacity (ΔC_p_) and molecular homology model building suggest a pronounced contribution of desolvation of non-polar groups upon binary complex formation. Thermodynamic parameters were deconvoluted into hydrophobic and vibrational contributions upon *Mt*SK:SKH binary complex formation. Data for the number of protons exchanged during this bimolecular interaction are interpreted in light of a structural model to try to propose the likely amino acid side chains that are the proton donors to bulk solvent following *Mt*SK:SKH complex formation.

## Introduction

Tuberculosis (TB), owing to *Mycobacterium tuberculosis* infection, still remains as a major global health problem. Approximately 9 million new cases of TB are detected each year, and close to 2 million people die from the disease [1]. In 2008, it has been estimated that 390 000–510 000 cases of multidrug-resistant tuberculosis (MDR-TB, which is defined as TB caused by strains of *M. tuberculosis* that are resistant to at least isoniazid and rifampicin) emerged globally [2]. In 2008, MDR-TB caused an estimated 150 000 deaths. It has also been reported that 5.4% of MDR-TB cases were found to have extensively drug-resistant tuberculosis (XDR-TB, which is defined as MDR-TB plus resistance to a fluoroquinolone and at least one second-line injectable agents: amikacin, kanamycin and/or capreomycin) [2]. In addition, TB cases due to infection with totally drug-resistant strains (TDR-TB) have been reported [3]. The emergence of drug-resistant strains of *M. tuberculosis* has thus highlighted the need for the development of new therapeutic strategies to combat TB.

Rational inhibitor design relies on mechanistic and structural information on the target enzyme. Enzymes offer unique opportunities for drug design that are not available to cell surface receptors, nuclear hormone receptors, ion channel, transporters, and DNA [4]. It has been pointed out that one of the lessons to be learned from marketed enzyme inhibitors is that the most potent and effective inhibitors take advantage of enzyme chemistry to achieve inhibition [5]. Moreover, the recognition of the limitations of high-throughput screening approaches in the discovery of candidate drugs has rekindled interest in rational design methods [6]. Accordingly, mechanistic analysis should always be a top priority for enzyme-targeted drug programs aiming at the rational design of potent enzyme inhibitors. Moreover, targets that are both essential to survival of, and exclusive to, *M. tuberculosis* are particularly promising as their inhibition could lead to the development of non-toxic drugs to the human host and having effective killing effect on the pathogen [7].

The biosynthesis of aromatic rings from carbohydrate precursors involves a range of chemical transformations that together constitute the shikimate pathway; through seven enzymatic steps, phosphoenolpyruvate (PEP) and D-erythrose 4-phosphate (E4P) are condensed to the branch point compound chorismate (endproduct), which leads to several additional terminal pathways [7], [8]. The shikimate pathway is essential in algae, higher plants, fungi, bacteria, apicomplexan parasites and sea anemone, but absent from humans [7]–[16]. The mycobacterial shikimate pathway (the main trunk) leads to the biosynthesis of chorismic acid, which is converted by five distinct enzymes to prephenate (precursor of phenylalanine and tyrosine), anthranilate (precursor of tryptophan), aminodeoxychorismate (precursor of *para*-aminobenzoic acid -PABA – which, in turn, leads to tetrahydrofolate synthesis), *para*-hydroxybenzoic acid (precursor of ubiquinone or Coenzyme Q), and isochorismate (common precursor of naphtoquinones, menaquinones and mycobactins) [7].

**Figure 1:**
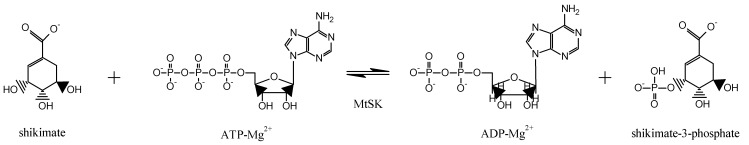
Shikimate Kinase catalyzed phosphoryl transfer from ATP to C3 hydroxyl group of shikimate (SKH), yielding shikimate 3-phosphate (S3P) and ADP.

The *aroK*-encoded *M. tuberculosis* Shikimate Kinase (*Mt*SK; EC 2.7.1.71), the fifth enzyme of the pathway, catalyzes a phosphoryl transfer from ATP to the carbon-3 hydroxyl group of shikimate (SKH; [3*R*-(3α,4α,5β)]3,4,5-trihydroxy-1-cyclohexene-1-carboxylic acid) forming shikimate 3-phosphate (S3P) (**Fig. 1**). Disruption of *aroK* gene has demonstrated that *Mt*SK, and thus the common aromatic biosynthesis pathway, is essential for the viability of *M. tuberculosis* [17]. We have previously reported cloning and expression in *Escherichia coli* of recombinant *Mt*SK in functional form [18], thereby confirming the correct *in silico* assignment to the structural gene encoding this protein. Our research group [19], [20] and others [21]–[23] have reported crystal structure determinations of *Mt*SK. Three functional motifs of nucleotide-binding enzymes were recognizable in *Mt*SK, including a Walker A-motif (that forms the phosphate-binding loop; P-loop), a Walker B-motif, and an adenine-binding loop. *Mt*SK belongs to the family of nucleoside monophosphate (NMP) kinases, which are composed of three domains: (1) the CORE domain containing the five stranded parallel β-sheet and the P-loop (residues 9–17), which forms the binding site for nucleotides; (2) the LID domain (residues glycine-112 to aspartate-124), which closes over the active site and has residues that are essential for the binding of ATP; and (3) the NMP-binding domain (residues threonine-33 to glutamate-61; also known as SB domain in *Mt*SK), which functions to recognize and bind shikimate [19]–[22]. More recently, based on an analysis of global movements upon ligand binding, it has been proposed that *Mt*SK is comprised of four domains [23]: (1) the ESB domain (extended SB; residues 32–93); (2) the nucleotide-binding (NB) site that includes the P-loop (Walker A motif, residues 9–17), the AB-loop (residues 148–155), and the segment of 101–110; (3) the LID domain (residues 112–124); and (4) the Reduced Core (RC) domain. A characteristic feature of NMP kinases is that they undergo large conformational changes during catalysis [24].

Based on a series of high-resolution crystal structures of *Mt*SK in apo form and as binary and ternary complexes, it has been proposed that the enzyme mechanism is random sequential binding of SKH and ATP (adenosine 5'-triphosphate), and release of ADP (ADP, adenosine 5′-diphosphate) product is followed by S3P to generate free enzyme [23]. However, no description of *Mt*SK enzyme mechanism in solution has, to the best of our knowledge, ever been described. Here we present purification of recombinant *Mt*SK to homogeneity, mass spectrometry analysis, N-terminal amino acid sequencing, and oligomeric state determination of the recombinant protein. We also present true steady-state kinetic parameters determination, and ligand binding by fluorescence spectroscopy and isothermal titration calorimetry (ITC) data. These data demonstrate that the chemical reaction catalyzed by monomeric *Mt*SK follows a random order mechanism of substrate binding, and that S3P product is released first followed by ADP dissociation to yield free enzyme. The ITC results provided the thermodynamic signatures of non-covalent interactions to the binding processes. In addition, we showed that there is a positive free energy coupling of 3.2 kJ mol^−1^ for SKH binding to *Mt*SK:Mg^2+^ATP binary complex. Accordingly, ATP appears to display negative cooperativity for SKH binding. Based on experimental evidence, we propose that *Mt*SK would more appropriately be described as an *aroL*-encoded type II shikimate kinase. We also present studies of the temperature dependence of thermodynamic parameters for SKH interaction with *Mt*SK. The change in constant pressure heat capacity (ΔC_p_) on going from free to bound states was evaluated and molecular homology model building was carried out to try to correlate complex formation with burial of surface area. Attempts were also made to deconvolute the thermodynamic parameters into hydrophobic and vibrational components. Determination of changes in binding enthalpy (ΔH) was carried out in the presence of buffers with different enthalpies of ionization. These ITC results showed that *Mt*SK:SKH binary complex formation is accompanied by release of protons to the bulk solvent. Based on structural information, we suggest that the δ-guanidinium groups of Arg58 and/or Arg136 are the likely sources of proton released into solution upon binary complex formation. Understanding the mode of action of *Mt*SK will inform us on how to better design inhibitors targeting this enzyme with potential therapeutic application in TB chemotherapy. The results here presented may also help chemical biologists to design function-based chemical compounds to carry out either loss-of-function (inhibitors) or gain-of-function (activators) experiments to reveal the biological role of *Mt*SK in the context of whole *M. tuberculosis* cells. Accordingly, it is hoped that the results here described may be useful to the rational design of anti-TB agents and that they may contribute to our understanding of the biology of *M. tuberculosis*.

## Materials and Methods

### Purification of *M. tuberculosis* Shikimate Kinase (MtSK)

The recombinant enzyme was expressed in *Escherichia coli* BL21 (DE3) host cells as previously described [18]. Approximately 6 g of cells were suspended in 24 mL of Tris-HCl (tris(hydroxymethyl)aminomethane) 50 mM pH 7.6, disrupted by sonication, and the cell debris removed by centrifugation (48,000 *g* for 60 min). MgCl_2_ was added to the supernatant to a final concentration of 10 mM followed by addition of 1 mg of DNAse, stirred for 30 min at 4°C, and centrifuged (10,000 *g* for 30 min). Interestingly, addition of MgCl_2_ resulted in precipitation of *Mt*SK whereas a number of proteins remained in the supernatant. Accordingly, this step served two purposes in the purification protocol: lysis of DNA by DNAse, and *Mt*SK precipitation. The pellet was suspended in Tris-HCl 50 mM pH 7.6 containing KCl 500 mM, and centrifuged (10,000 *g* for 15 min). This solution was concentrated down to approximately 20 mL, and 20 mL of Tris-HCl 50 mM pH 7.6 containing KCl 500 mM and (NH_4_)_2_SO_4_ 2 M was added, resulting in a solution containing 1 M (NH_4_)_2_SO_4_. This solution was clarified by centrifugation. The supernatant was loaded on a Phenyl Sepharose 16/10 column (GE healthcare) previously equilibrated with buffer A (Tris-HCl 50 mM, KCl 500 mM, (NH_4_)_2_SO_4_ 1 M, pH 7.6) and the adsorbed material eluted with a linear gradient of buffer B (Tris-HCl 50 mM, KCl 500 mM, pH 7.6) at 1 mL min^−1^. The protein fractions containing the *Mt*SK were pooled and loaded on a Sephacryl S-100 HR column (GE Healthcare) and isocratically eluted with buffer B at 0.25 mL min^−1^. The fractions containing homogeneous recombinant *Mt*SK were pooled, and stored in 85% (NH_4_)_2_SO_4_. Protein expression, purification and apparent homogeneity of recombinant *Mt*SK was confirmed by 12% sodium dodecyl sulfate-polyacrylamide gel eletrophoresis (SDS-PAGE) stained with Coomassie Brilliant Blue [25]. Protein concentration was determined by the method of Bradford *et al*. [26] using the Bio-Rad protein assay kit and bovine serum albumin as standard (Bio-Rad Laboratories).

### Electrospray Ionization Mass Spectrometry (ESI-MS) Analysis

The subunit molecular mass of recombinant protein preparation was assessed by ESI-MS, employing some adaptations made to the system described by Chassaigne and Lobinski [27]. Samples were analyzed on a triple quadrupole mass spectrometer (model QUATTRO II) equipped with a standard electrospray (ESI) probe (Micromass, Altrincham, United Kingdom), and adjusted to a flow rate of *ca*. 250 µL min^−1^. The source temperature (80°C) and needle voltage (3.6 kV) were maintained constant throughout the experimental data collection, applying a drying gas (nitrogen) flow of 200 L h^−1^ and a nebulizer gas flow of 20 L h^−1^. The mass spectrometer was calibrated with intact horse heart myoglobin and its typical cone-voltage induced fragments. The subunit molecular mass of recombinant *Mt*SK was determined by adjusting the mass spectrometer to give a peak with at half-height of 1 mass unit, and the sampling cone-to-skimmer lens voltage controlling the transfer of ions to the mass analyzer was set to 38 V. About 50 pmol (10 µL) of each sample was injected into the electrospray transport solvent. The ESI spectrum was obtained in the multichannel acquisition mode, scanning from 500 to 1,800 *m*/*z* at a scan time of 7 s. The mass spectrometer is equipped with MassLynx and Transform software for data acquisition and spectrum handling.

### N-terminal Amino Acid Sequencing

The N-terminal amino acid residues of homogeneous recombinant MtSK were identified by automated Edman degradation sequencing method [28] using PPSQ 21A gas-phase sequencer (Shimadzu).

### Oligomeric State Determination

The oligomeric state of homogeneous *Mt*SK was determined by size exclusion liquid chromatography on Superdex 200 (HR 10/30) column (GE Healthcare). The column was pre-equilibrated with 50 mM Tris-HCl pH 7.6 containing 200 mM NaCl at a flow rate of 0.4 mL min^−1^ (4°C), with UV detection at 280 nm. The calibration curve was constructed employing the following protein standards: ribonuclease A (13.7 kDa), quimotripsinogen (25 kDa), ovalbumine (43 kDa), albumine (67 kDa), aldolase (158 kDa), catalase (232 kDa), and ferritine (440 kDa). The elution volumes (*V*_e_) of calibration proteins were used to calculate their corresponding partition coefficient (*K*_av_, Eq. 1). Blue dextran was utilized for determination of the void volume (*V*_o_). *V*_t_ is the total bead volume of the column. The *K*_av_ values for the standards were plotted versus the logarithm of their corresponding molecular masses. A volume of 100 µL of recombinant protein was loaded on the gel filtration column to obtain *V*_e_ for *Mt*SK.
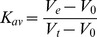
(1)


### Steady-state Kinetics

Recombinant *Mt*SK enzyme activity was assayed in the the forward direction by coupling the ADP product to the pyruvate kinase (PK; EC 2.7.1.40) and lactate dehydrogenase (LDH; EC 1.1.1.27) reactions following the protocol described by Millar *et al.* [29]. Shikimate-dependent oxidation of NADH (nicotinamide adenine dinucleotide) was continuously monitored at 340 nm (ε = 6.22×10^3^ M^−1^ cm^−1^). All reactions were carried out at 25°C and initiated with addition of recombinant *Mt*SK (1 µg mL^−1^). The assay mixture contained 100 mM Tris–HCl buffer, pH 7.6, 100 mM KCl, 5 mM MgCl_2_, 1.5 mM PEP, 0.2 mM NADH, 6 U mL^−1^ PK, and 5 U mL^−1^ LDH. Initial steady-state rates were calculated from the linear portion of the reaction curve under experimental conditions in which less than 5% of substrate was consumed. True steady-state kinetics parameters were determined from initial velocity measurements at varying concentrations of SKH (37–4800 µM) at varied-fixed ATP concentrations (9–1200 µM).

Values of the steady-state kinetics parameters and their respective errors were obtained by fitting the data to the appropriate equations using the non-linear regression function of SigmaPlot 9.0 (SPSS, Inc). Hyperbolic saturation curves of initial rate data at single concentration of the fixed substrate and varying concentrations of the other were fitted to the Michaelis-Menten equation (Eq. 2) [30], [31], in which *v* is the initial velocity, *V* is the apparent maximum initial velocity, *A* is the varying substrate concentration and *K* represents the apparent Michaelis-Menten constant.
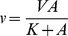
(2)


The family of lines intersecting to the left of the *y*-axis in double-reciprocal plots was fitted to Eq. 3, which describes a mechanism involving ternary complex formation and a sequential substrate binding [30], [31].
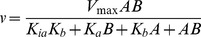
(3)


For Eq. 3, *v* is the initial velocity (as for Eq. 2), *V_max_* is the true maximum initial velocity, *A* and *B* are the concentrations of the substrates, *K*_a_ and *K*_b_ are their respective Michaelis constants, and *K*_ia_ is the dissociation constant for enzyme-substrate A binary complex formation.

### Equilibrium Fluorescence Spectroscopy

*Mt*SK intrinsic protein fluorescence measurements were carried out to both determine the order of substrate/product addition/dissociation on/from the catalytic site and distinguish the enzyme kinetic mechanism. As *Mt*SK has no tryptophan residues, changes in protein tyrosine fluorescence (the polypeptide chain of *Mt*SK has 3 Tyr residues) upon ligand binding were monitored. Fluorescence measurements were carried out in a RF-5301 PC Spectrofluorophotometer (Shimadzu) at 25°C. Excitation wavelength was 280 nm and emission spectra were collected from 300 to 500 nm. The maximum values of fluorescence intensity at 315 nm values were plotted as a function of increasing ligand concentration. Excitation and emission slits were 5 nm. As protein tyrosine typically has a low sensitivity (low molar absorption coefficient and low fluorescence quantum yield), *Mt*SK concentration for binding experiments was 10 µM. Fluorescence titrations of binary complex formation were carried out by making microliter additions of the following compounds to 2 mL solution containing 10 µM *Mt*SK in 100 mM Tris-HCl, 100 mM KCl, 5 mM MgCl_2_, pH 7.6∶120 mM SKH stock solution (10–1100 µM final concentration); 120 mM S3P stock solution (59.97–952.3 µM final concentration). Control experiments were performed in the same experimental conditions except that no substrate was added, and these values were subtracted from those obtained in the presence of substrate. However, owing to a large inner filter effect, ATP and ADP binding to free *Mt*SK could not be determined by fluorescence spectroscopy.

Equilibrium fluorescence spectroscopy data were fitted to Eq. 4, in which *F* is the observed fluorescence, *F*_0_ is the initial fluorescence, *F*_∞_ is the maximum change in fluorescence at saturating ligand (L) concentration, and *K*_D_ represents the equilibrium dissociation constant for protein:ligand binary complex formation.
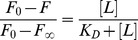
(4)


### Isothermal Titration Calorimetry (ITC)

ITC experiments were carried out using an iTC_200_ Microcalorimeter (Microcal, Inc., Northampton, MA). The equipment’s sample cell volume is 200 µL and syringe final volume is 39 µL. Calorimetric experiments were performed with either substrates (SKH and ATP) or products (S3P and ADP) at 298.15 K. The reference cell (200 µL) was loaded with water during all experiments and the sample cell (200 µL) was filled with *Mt*SK at 100 µM concentration for ATP and ADP binding experiments, and at 130 µM for SKH and S3P binding experiments. The injection syringe (39 µL) was filled with substrates or products at different concentrations: ATP and ADP at 6 mM, and SKH and S3P at 4.2 mM. Owing to the large enthalpy of ionization of Tris buffer [32] that we employed in steady-state kinetics and fluorescence spectroscopy, all ITC measurements were carried out in HEPES (*N*-2-hydroxyethylpiperazyne-*N’*-2-ethanesulfonic acid) 50 mM, KCl 50 mM and MgCl_2_ 5 mM, pH 7.6. The binding reaction started with one injection of 0.5 µL of ligand to prevent artifacts, followed by 17 injections of 2.26 µL at intervals of 180 s, reaching a final volume 39 µL with a stirring speed of 500 RPM. To evaluate the temperature dependence of the binding enthalpy of *Mt*SK:SKH binary complex formation, the complex formation was investigated at several temperatures (273.15–313.15 K), in the same conditions as at 298.15 K (Eq. 5). Furthermore, binding experiments were carried out at pH 7.6 (298.15 K) in buffers with different enthalpies of ionization (ΔH_ion_) (Pipes 2.68 kcal mol^−1^; Hepes 4.88 kcal mol^−1^; Imidazole 8.76 kcal mol^−1^) [33] as a method to determine any proton exchange between the binary complex and buffer (N_H+_), the intrinsic enthalpy (ΔH_int_) and a possible p*K*_a_ shift of an ionizable group in the binding pocket (Eq. 6 and Eq. 7). N_H+_ represents the number of protons exchanged in the process of complex formation, and a negative value for N_H+_ represents either the number of protons taken up by the buffer or released by the protein-ligand complex. For the Henderson-Hasselbalch equation (Eq.7), [HA] represents the concentration of acid and [A^-^] the concentration of its conjugate base. The heat variation was monitored inside the cell allowing determination of binding enthalpy of the process (ΔH) and the equilibrium association constant (K_a_). All enthalpy values for binding reactions were exothermic. Control titrations were performed to subtract the heats of dilution and mixing for each experiment.

The Gibbs free energy (ΔG) of binding was calculated using the relationship described in Eq. 8, in which R is the gas constant (8.314 J K^−1^ mol^−1^; 1.987 cal K^−1^ mol^−1^), T is the temperature in Kelvin (T = °C +273.15), and K_a_ is the association constant at equilibrium. The entropy of binding (ΔS) can also be determined by this mathematical formula. Single set of sites model was utilized to determine the binding and thermodynamics constants. The initial value for n was fixed as 1 since *Mt*SK is monomeric in solution, and estimates for K_a_, and ΔH parameters were refined by standard Marquardt nonlinear regression method provided in the Origin 7 SR4 software.
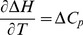
(5)

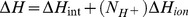
(6)

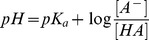
(7)

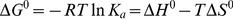
(8)


### Molecular Homology Model Building

To try to understand the protein conformational variation and non-covalent interactions upon SKH binding to *Mt*SK, bioinformatics tools were used to reconstruct and analyze the structures. The missing residues in the structures of apo *Mt*SK (PDB code 2IYT) and *Mt*SK in complex with S3P and ADP (PDB code 2IYZ) were reconstructed using Modeller [34] and a total of 100 models were generated. The pose of SKH was based on that of S3P present in the crystal. Each model of the 100 complex models was minimized using the conjugate gradient algorithm (300 iterations) in Gromacs [35]. The polar (ΔASA_pol_) and non-polar (ΔASA_non-pol_) solvent accessible surface areas (ASA) were calculated using an analytical method based on Voronoi surfaces [36]. To account for the dynamic nature of protein structures, ASA values were calculated for each individual complex and their average values used in the analysis.

## Results and Discussion

### Recombinant *MtSK* Protein Purification

**Figure 2:**
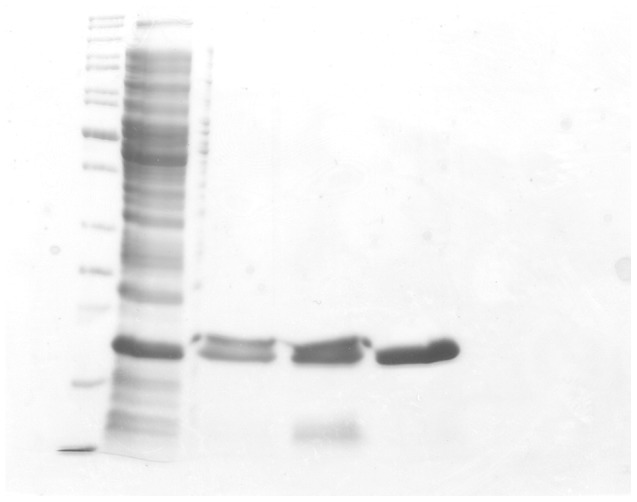
12% SDS-PAGE analysis of pooled fractions of Mt*SK* for each purification step. Lane 1, Protein Molecular Weight Marker (Fermentas); lane 2, soluble *E. coli* BL21 (DE3) [pET-23a(+)::aroK] extract; lane 3, Soluble proteins after 10 mM MgCl_2_ precipitation step; lane 4, Phenyl Sepharose 16/10; and lane 5, Sephacryl S-100 HR.

Recombinant *Mt*SK was purified to homogeneity using a three-step protein purification protocol comprising a crude extract precipitation, a hydrophobic chromatographic step (Phenyl Sepharose) followed by a gel filtration column (Sephacryl S-100) (**Fig. 2**). The protein precipitation of the crude extract with MgCl_2_ at a final concentration of 10 mM was efficient at precipitating *Mt*SK whereas a number of contaminants remained in the supernatant. It has been pointed out that direct ion-macromolecule interactions as well as interactions with water molecules in the first hydration shell of macromolecules appear to play a central role to Hofmeister effects [37]. The Hofmeister series ranks the relative influence of ions on the physical behavior of a wide variety of aqueous processes ranging from colloidal assembly to protein folding. Usually, the specific ion effects of the Hofmeister series are more pronounced for anions than for cations [37]. The Cl^-^ anion is situated in the borderline between kosmotropes (“water structure makers”, strongly hydrated, stabilizing and salting-out effects on proteins) and chaotropes (“water structure breakers”, destabilize folded proteins and have salting-in effects on proteins) species. The Mg^2+^ ion has chaotropic (salting-in) effect. It is thus somewhat puzzling the salting-out effect of 10 mM MgCl_2_ on *Mt*SK. However, it has recently been pointed out that the transport number of Mg^2+^ cation is higher than that for the other common biological cations, and the solvent exchange rate is over 3 orders of magnitude less than that for other common cations [38]. It would thus imply that Mg^2+^ cation would have a significant but largely unstudied effect on ordering of solvent and molecules in solution [38]. Notwithstanding, it is not warranted to advance any definite explanation as regards the MgCl_2_ salting-out effect on *Mt*SK. This protein precipitation step was followed by two chromatographic steps, namely, a hydrophobic followed by a size-exclusion column, yielding approximately 20 mg of functional homogenous *Mt*SK per 1.5 L of cell culture. The homogeneous recombinant *Mt*SK was stored in 85% (NH_4_)_2_SO_4_ at 4°C with no loss of activity.

### Electrospray Ionization Mass Spectrometry (ESI-MS) Analysis

A value of 18,451 Da for the subunit molecular mass of recombinant *Mt*SK protein was determined by ESI-MS. This result is consistent with post-translational removal of N-terminal methionine residue (131.2 Da) from the full-length gene product (predicted mass, 18,583 Da). The ESI-MS result also revealed no peak at the expected mass of both *aroK*-encoded Shikimate Kinase I (19,526 Da) and *aroL*-encoded Shikimate Kinase II (18,998 Da) from *E. coli* SK [39], thus providing support for the identity of purified recombinant protein.

### N-terminal Amino Acid Sequencing

The first 8 N-terminal amino acid residues of recombinant *Mt*SK were identified as APKAVLGL by the Edman degradation sequencing method. This result unambiguously identifies the purified protein as *Mt*SK, since the N-terminal amino acid sequence of *aroK*-encoded Shikimate Kinase I and *aroL*-encoded Shikimate Kinase II from *E. coli* are, respectively, MAEKRNIFLV and MTQPLFLIGP. The Edman degradation result also confirms removal of the N-terminal methionine residue, and is in agreement with no observation of N-terminal methionine in the crystal structure of *Mt*SK:MgADP:SKH ternary complex [19]. The excision of N-terminal methionine is a common type of post-translational modification process that occurs in protein synthesized in the cytoplasm of prokaryotic cells. The cleavage of the initiator methionine is usually directed by the penultimate amino acid residues with the smallest side chain radii of gyration (Gly, Ala, Ser, Thr, Pro, Val, and Cys) [40]. Removal of N-terminal methionine from recombinant *Mt*SK polypeptide chain conforms to this rule since alanine is the penultimate amino acid residue.

### Oligomeric State Determination

A value of 20.7±0.5 kDa for the molecular mass of homogeneous recombinant *Mt*SK was estimated by gel filtration chromatography (data not shown). This result demonstrates that *Mt*SK is a monomer in solution, since ESI-MS analysis suggested a value of 18,451 Da for the subunit molecular mass of the recombinant protein.

### Steady-state Kinetics

**Figure 3:**
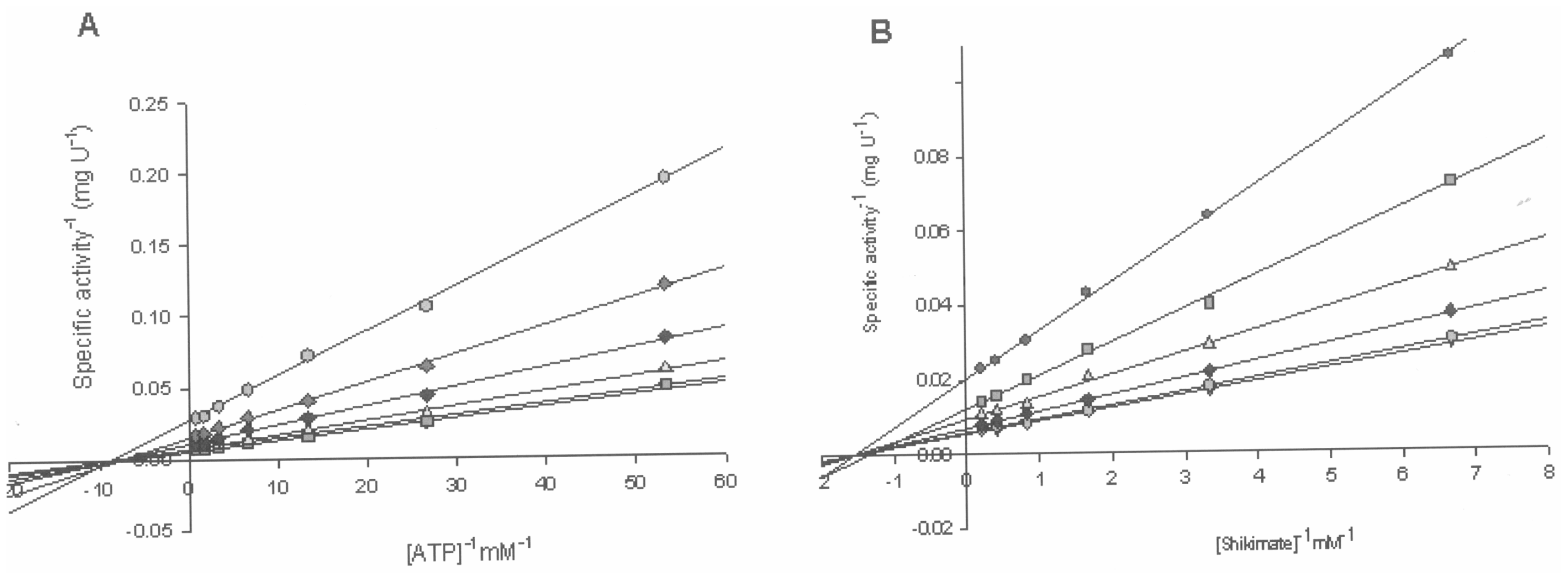
Double-reciprocal plots for steady-state kinetics of *Mt*SK using either ATP (A) or SKH (B) as the variable substrate. Each curve represents varied-fixed levels of the co-substrate, ranging from 37 to 4800 µM for SKH and from 9 to 1200 µM to ATP.

Hyperbolic initial velocity as a function of substrate concentration (either ATP or SKH) was plotted as a linear function of reciprocal of initial velocity against the reciprocal of substrate concentration (double-reciprocal or Lineweaver-Burk plot). These data allow both determination of true steady-state kinetic parameters and a proposal for *Mt*SK enzyme mechanism. The double-reciprocal plots showed a family of lines intersecting to the left of the *y*-axis (**Fig. 3**), which is consistent with ternary complex formation and a sequential mechanism. This pattern of lines rules out ping-pong (parallel lines), steady-state random (that gives non-linear reciprocal plots), and rapid-equilibrium ordered (one of the family of lines should cross at a single value on the *y*-axis) mechanisms. However, the double-reciprocal plots alone cannot distinguish between rapid-equilibrium random and steady-state compulsory ordered bi bi mechanisms. The double-reciprocal data were fitted to the equation for a sequential initial velocity pattern (Eq. 3), yielding the following values for the true steady-state kinetic parameters (Table 1): *k*_cat_ = 60 (±8) s^−1^, *K_MgATP_* = 112 (±4) µM, *K_SKH_* = 650 (±28) µM, *k*_cat_/*K_MgATP_* = 5.4 (±0.7)×10^5^ M^−1^ s^−1^, and *k*_cat_/*K_SKH_* = 0.9 (±0.1)×10^5^ M^−1^ s^−1^. *E. coli* has two Shikimate Kinase enzymes: *aroK*-encoded SK I and *aroL*-encoded SK II [41]. The *K_SKH_* value for *E. coli* SK I (20 mM) is larger than the value for *E. coli* SK II (200 µM) (**Table 1**). Although the complete sequencing of *M. tuberculosis* H37Rv genome has identified by sequence homology the presence of *aroK*-encoded SK I [42], the kinetic data presented here and elsewhere [43] show that *Mt*SK would more appropriately be described as an *aroL*-encoded type II enzyme. In addition, although the steady-state kinetic parameters are in good agreement with ones previously reported [43], no description of *Mt*SK enzyme mechanism was presented, indicating a need of a more complete enzymatic study, as described here. To distinguish between random and compulsory ordered bi bi mechanisms, substrate(s) and product(s) binding experiments were carried out as described below.

**Table 1:** Steady-state kinetics constants of Shikimate Kinases (SKs) from different organisms.

	*Mt*SK^1^	*Mt*SK^2^	*E. coli* SKI^2^	*E. coli* SKII^2^	EcSK^3^
k_cat_(s^−1^)	60 (±8)	44 (±2)		32	35
*K*_MgATP_ (µM)	112 (±4)	83 (±4)		160	620
*K*_SKH_ (µM)	650 (±28)	410 (±20)	20×10^3^	200	310
*k*_cat_/K_MgATP_ (M^−1^ s^−1^)	5.4 (±0.7)×10^5^	5.3×10^5^		2.0×10^5^	5.6×10^4^
*k*_cat_/K_SKH_ (M^−1^ s^−1^)	0.9 (±0.1)×10^5^	1.1×10^5^		1.6×10^5^	1.1×10^5^

1 Results described here.

2 adapted from reference [43].

3 SK from *Erwinia chrysanthemi*. (values taken from reference [54]).

### Equilibrium Fluorescence Spectroscopy

**Figure 4:**
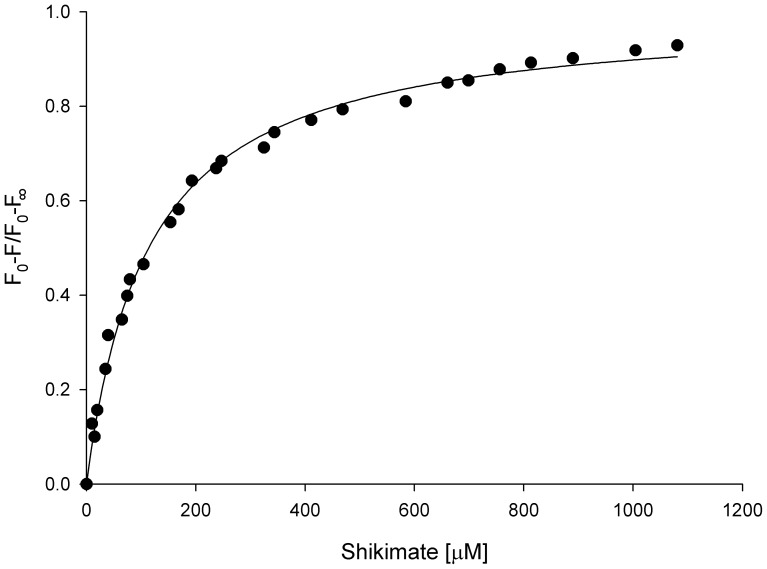
Fluorescence spectroscopy of the equilibrium binding of SKH to *Mt*SK, measuring the quench in intrinsic protein fluorescence upon ligand binding andp plotting the relative fluorescence change as a function of SKH concentration.

In equilibrium binary complex formation experiments, there was a quench in the intrinsic *Mt*SK protein fluorescence upon binding of SKH, however no fluorescence variation was observed due to S3P presence. Titration of *Mt*SK with SKH was hyperbolic (**Fig. 4**), and fitting the data to Eq. 4 yielded a value of 113 (±4) µM for the equilibrium dissociation constant of SKH (*K*_D_). No change in protein fluorescence could be observed for S3P binding to free *Mt*SK (data not shown). However, it cannot be assumed that no *Mt*SK:S3P binary complex was formed because S3P binding to free enzyme may result in no change in protein fluorescence. In addition, as previously mentioned, ATP and ADP binding to *Mt*SK could not be assessed by fluorescence spectroscopy due to large inner filter effects. Accordingly, ITC experiments were carried out.

### Isothermal Titration Calorimetry (ITC)

**Figure 5:**
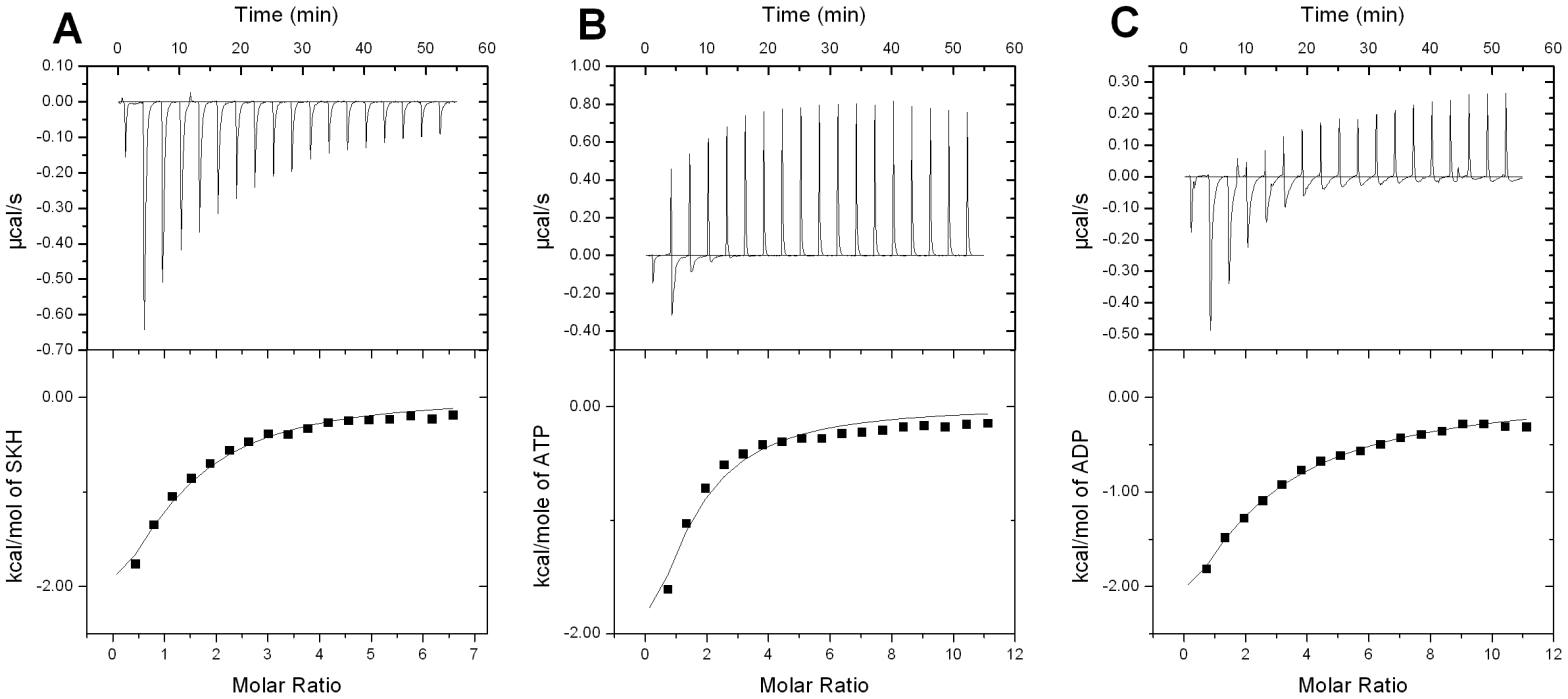
Isothermal titration calorimetry (ITC) analysis of either SKH (A), Mg^2+^ATP (B), or Mg^2+^ADP (C) binding to *Mt*SK. The top graphs show raw data of the heat pulses resulting from titration of *Mt*SK (100 µM for ATP and ADP, and 130 µM for SKH and S3P) in the calorimetric cell with one injection of 0.5 µL of ligand followed by 17 injections of 2.26 µL injection. The injection syringe (39 µL) contained either ATP or ADP at 6 mM, or SKH and S3P at 4.2 mM. The bottom graphs show the integrated heat pulses, normalized per mol of injectant as a function of the molar ratio (ligand/*Mt*SK concentration). These binding curves were best fitted to a single set of sites model equation.

ITC measurements were carried out to both determine the order, if any, of addition of substrate and the order of product release to yield free enzyme. In agreement with fluorescence spectroscopy results, no binding of S3P to free enzyme could be detected by ITC measurements (data not shown). On the other hand, ITC measurements showed binding of SKH, Mg^2+^ATP and Mg^2+^ADP to free *Mt*SK enzyme (**Fig. 5**, **Table 2**). These results support a mechanism in which binding of substrates (SKH and ATP) to *Mt*SK is random, and S3P product release is followed by ADP dissociation to yield free enzyme. The value for the SKH equilibrium dissociation constant determined at 298.15 K by fluorescence spectroscopy (113 µM) is in good agreement with the value determined from ITC data (181 µM) at the same temperature. As the double-reciprocal plots of initial velocity measurements could not distinguish between rapid-equilibrium random and steady-state compulsory ordered bi bi mechanisms, fluorescence spectroscopy and ITC results indicate that the bi bi mechanism of *Mt*SK is rapid-equilibrium random order of substrate addition. Based on a series of crystal structures, it has been proposed that substrate binding to *Mt*SK is random [23], in agreement with the results presented here. However, whether an enzyme mechanism is rapid equilibrium or steady state cannot be identified by crystal structure determinations. In addition, crystal structure analysis suggested that ADP product release is followed by S3P to generate free enzyme [23]. Here we demonstrate that S3P product is released first, followed by dissociation of ADP from *Mt*SK:ADP binary complex to generate free enzyme for the next round of catalysis.

**Table 2:** Thermodynamics parameters of formation of binary complexes between *Mt*SK and substrate(s) or product(s).

	K_D_ (µM)	ΔH (kcal mol^−1^)	ΔS (cal mol^−1^ K^−1^)	ΔG (kcal mol^−1^)	-TΔS (kcal mol^−1^)
SKH _283.15 K, Hepes_	45 (±13)	0.66 (±0.04)	22.2 (±6.5)	−5.6 (±1.6)	−6.2 (±1.8)
SKH _298.15 K, Hepes_	181 (±15)	−4.6 (±0.2)	1.8 (±0.2)	−5.1 (±0.4)	−0.54 (±0.05)
SKH _313.15 K, Hepes_	235 (±9)	−8.9 (±0.1)	−11.9 (±0.4)	−5.2 (±0.2)	3.7 (±0.1)
SKH _298.15 K, Pipes_	135 (±8)	−3.5 (±0.1)	5.7 (±0.3)	−5.2 (±0.3)	−1.7 (±0.2)
SKH _298.15 K, Imidazole_	396 (±47)	−6.4 (±0.4)	−6.0 (±0.7)	−4.6 (±0.2)	1.8 (±0.1)
ATP	196 (±29)	−5.1 (±0.3)	−0.15 (±0.02)	−5.1 (±0.8)	0.044 (±0.007)
ADP	562 (±20)	−12.4 (±0.2)	−27 (±1)	−4.4 (±0.2)	7.9 (±0.2)

K_D_ represents the equilibrium dissociation constant, ΔH is the binding enthalpy, ΔS is the binding entropy, ΔG is the Free Gibbs energy, and –TΔS is the negative term for temperature (in Kelvin) times binding entropy.

The ITC results showed significant heat changes upon ligand (SKH, ATP, or ADP) binding to free *Mt*SK enzyme, thereby providing thermodynamic signatures of non-covalent interactions to each binding process. Observed enthalpies arise largely as a result of changes in interatomic interactions (e.g., hydrogen bonds and/or van der Waals interactions), in which the sign indicates whether there is a net favourable (negative Δ*H*) or unfavourable (positive Δ*H*) redistribution of the network of interactions between the reacting species (including solvent) [44]. Hydrophobic interactions are related to the relative degrees of disorder in the free and bound systems and thus these interactions are reflected in the entropy change. The release of “bound” water molecules from a surface to the bulk solvent is usually a source of favourable entropy (positive Δ*S*). A reduction in conformational states in either ligand or protein upon binary complex formation is entropically unfavourable (negative Δ*S*) [44].

The ITC data for Mg^2+^ADP binding to *Mt*SK suggest that it is accompanied by a favourable redistribution of H-bonds and/or van der Waals interactions, and a large entropically unfavorable contribution, resulting in a large value for the equilibrium dissociation constant (**Table 2**). It is thus tempting to suggest that large *Mt*SK protein conformational changes occurring upon dissociation of Mg^2+^ADP from *Mt*SK:Mg^2+^ADP binary complex would regenerate free enzyme in a conformation that allows binding of substrate(s) to start a new cycle of catalysis. In addition, the large *K*_D_ value for ADP may avoid *Mt*SK:Mg^2+^ADP:SKH dead-end ternary complex formation, which would lock the enzyme active site in an inactive form. The ITC data for Mg^2+^ATP binding to *Mt*SK indicate that the molecular recognition process is accompanied by favourable redistribution of H-bonds and/or van der Waals interactions, and unfavourable entropic contribution (**Table 2**). The latter may reflect protein conformational changes upon Mg^2+^ATP binding. As pointed out above, *Mt*SK belongs to the family of NMP kinases, which are composed of CORE, LID and NMP-binding (SKH-binding or SB) domains. The LID domain closes the active site and has residues that are essential for ATP binding [19]–[23]. Moreover, NMP kinases undergo large conformational changes during catalysis [24]. It should be pointed out that a fourth domain has been proposed to be present in *Mt*SK structure, namely, the RC domain [23]. Interestingly, the dissociation constant value for binding of ATP (196 µM) is smaller than for ADP (562 µM). It may be accounted for by the direct interaction between the γ-phosphate of ATP and either Arg117 residue observed in the crystal structure of *Mt*SK:ATP binary complex or Lys15 that is part of the P-loop [23]. The unfavourable entropic contributions upon ATP and ADP binding to free *Mt*SK enzyme can thus be accounted for by protein conformational changes. The NMP-binding (or SB) domain of *Mt*SK functions to recognize and bind SKH [19]–[23]. The SKH substrate binding to free *Mt*SK is associated with favourable H-bonds and/or van der Waals interactions and a positive entropic contribution (**Table 2**). The latter may reflect release of “bound” water molecules either from substrate or from *Mt*SK active site to solvent. Since SKH is a hydrophilic molecule, with a logP of −2.22 and a logD at pH 7.4 of −5.1 the release of water molecules from the complex to the bulk is among a likely contribution to the favorable entropy of binding [45]. This favorable entropic contribution should however compensate for the unfavorable entropic contribution due to conformational changes known to occur upon SKH binding to *Mt*SK [19], [21]–[23]. The equilibrium constant for the intramolecular hydrolysis of bound ATP to bound ADP and phosphate at enzyme active sites is considerably larger than the equilibrium constant for ATP hydrolysis in solution [46]. The ITC data on SKH binding may indicate exclusion of water molecules from *Mt*SK active site to minimize ATP hydrolysis, in agreement with previous proposals [19]. Taken together, the ITC data may be reporting on protein conformational changes upon ATP or ADP binding, and on exclusion of water molecules from *Mt*SK active site upon SKH binding. These conclusions are in agreement with structural data on *Mt*SK that showed large rotational movement of the nucleotide binding domain upon ADP and ATP binding, bringing it closer to the SKH binding domain [23]. However, the ITC data on SKH at 298.15 K suggest that the unfavorable entropy due to rotational movements of the ESB domain and LID closure upon substrate binding to *Mt*SK [23] is likely to be cancelled out by the release of water molecules to bulk solvent.

**Figure 6:**
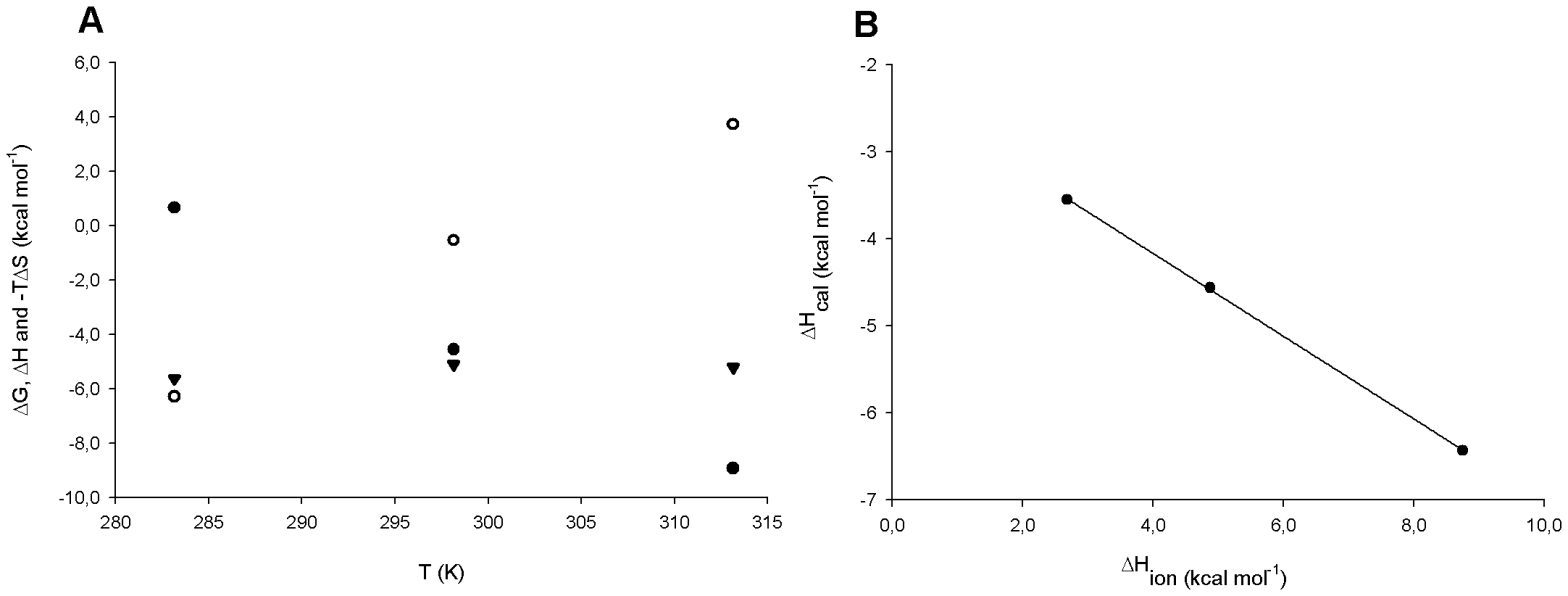
*Mt*SK:SKH thermodynamic binding parameters as a function of temperature (A) and binding enthalpy as a function of buffer ionization enthalpy at pH 7.6 (B). Figure 7A shows the ΔH (filled circles), -TΔS (open circles) and ΔG (inverted filled triangles) dependence on a temperature ranging from 10 to 40°C, which permits determination of a ΔC_p_ value of −320 (±16) cal mol^−1^ K^−1^. Figure 7B shows the dependence of observed enthalpy on buffer ionization enthalpy (ΔH_ion_) at 25 °C. Data fitting to Eq. 6 yielded a value of −0.47 for N_H+_ (number of protons exchanged during the binding process) and −2.2 kcal mol^−1^ for ΔH_int_ (intrinsic enthalpy).

The temperature dependence of thermodynamics parameters can point to the hydrophobic properties of the SKH interaction with *Mt*SK [45]. Changes in constant pressure heat capacity (ΔC_p_) on going from free to bound states have been used to correlate complex formation with burial of surface area [44], [45]. The data presented in **Fig. 6A** shows a graphical representation of observed ΔG, ΔH and -TΔS *versus* temperature for experiments performed at pH 7.6 in standard buffer (Hepes 50 mM, KCl 50 mM and MgCl_2_ 5 mM). A value of −320 (±16) cal mol^−1^ K^−1^ for ΔC_p_ (Eq. 5) of substrate binding was derived from the slope of the linear curve describing the changes in ΔH values as a function of increasing temperature (**Fig. 6A**). Previous works showed that the nature of a negative ΔC_p_ can be derived from difference in polar and non-polar surface areas exposed to the solvent [47], [48]. Since ΔC_p_ is strongly correlated with polar and non-polar surface exposed to the solvent [49], the variation of the surfaces were analyzed subtracting the solvent accessible surface areas (ASA) of free forms of SKH and *Mt*SK from *Mt*SK:SKH binary complex. Values of 440 Å^2^ and 1262 Å^2^ were estimated for, respectively, ΔASA_pol_ and ΔASA_non-pol_. These estimates suggest that desolvation of non-polar groups upon binary complex formation makes a more pronounced contribution to ΔC_p_ (negative) as compared to desolvation of polar groups (positive). This phenomenon can be addressed by the classical explanation that hydrophobic effect is due to the properties of solvent water in repelling non-polar molecules and groups [50]. Upon binary complex formation, the ΔASA_non-pol_ value indicates that there is a decrease in the number of non-polar groups exposed to the solvent. The negative value for ΔC_p_ could indicate a reduction in the number of hydration shells of non-polar groups, and ensuing decrease in entropy as temperature increases (positive value for -TΔS) and constant ΔG/ΔT (**Fig. 6A**). The nature of this interaction is temperature dependent [50], which is endothermic at low temperatures and exothermic at higher temperatures, as appears to be borne out by the results presented in **Fig. 6A**. A decrease in vibrational freedom upon binary complex formation and ensuing decrease in the energy of fluctuation between two states may also be invoked to explain the negative value for ΔC_p_. Notwithstanding, there are a number of contributing factors to ΔC_p_ values, including hydrogen bonding, electrostatics, protein conformational entropy and changes in equilibrium [49] that were not taken into account in our data analysis. The thermodynamic data in the temperature range studied show enthalpy-entropy compensation, resulting in temperature-independent ΔG values.

Gurney defined the ΔS as the sum of two components, the unitary entropy (ΔS_U_) and the cratic entropy (ΔS_C_ ) [51]. As pointed out by Irudayam and Henchman [52], the ΔS_C_ is a “controversial term whose interpretation and inclusion has been the subject of much debate”, since, after 59 years later Gurney paid attention to this term, a consensus about this theory has not been found. Sturtevant in 1977 [49] considered the ΔS_C_ in the formulation of his empirical method. A value of +7.9826 cal K^−1^ mol^−1^ [53] for the cratic contribution was used to correct the ΔS values found in this work to give ΔS_U_. Hydrophobic and vibrational components of thermodynamic parameters were achieved utilizing the empirical method developed by Sturtevant [49], in which the ΔS, ΔH and ΔC_p_ are deconvoluted in vibrational and hydrophobic contributions (**Table 3**). These results show that there is a strong hydrophobic contribution upon *Mt*SK:SKH binary complex formation, suggesting a decrease in the contact of the solvent with the apolar groups. In addition, there is an increase in magnitude of the vibrational contribution, which, as suggested by Sturtevant [49], may be ascribed to conversion of soft internal vibrational modes to stiffer modes upon ligand (SKH) binding. The crystal structure of *Mt*SK:SKH binary complex indicated that there appears to be a continuum of conformations, and that one of them showed no closure of the LID domain upon SKH binding [23], which would be consistent with no conformational changes upon SKH binding that would be entropically unfavorable and water release as the main contribution to the favorable entropy (positive Δ*S*). In addition, the results presented here (**Tables 2** **and** **3**) indicate that the unfavorable entropy due to rotational movements of the ESB domain and LID closure following SKH binding to *Mt*SK [23] is likely to be cancelled out by hydrophobic and vibrational contributions to binary complex formation.

**Table 3:** Hydrophobic and vibrational thermodynamic parameters of *Mt*SK:SKH binary complex formation.

T (K)	ΔH_H_(kcal mol^−1^)	ΔH_V_(kcal mol^−1^)	ΔS_H_(cal mol^−1^ K^−1^)	ΔS_V_(cal mol^−1^ K^−1^)	ΔC_pH_(kcal mol^−1^ K^−1^)	ΔC_pV_(kcal mol^−1^ K^−1^)	ΔG_H_(kcal mol^−1^ K^−1^)	ΔG_V_(kcal mol^−1^ K^−1^)
283.15	6.7	−6.0	72	−42	−278	−40	−13	5.9
298.15	5.2	−9.8	66	−65	−257	−61	−17	9.6
313.15	2.0	−11	65	−69	−252	−66	−18	10

ΔC_p_ represents the heat capacity; the H and V subscripts represent, respectively, the hydrophobic and vibrational contributions.

Ligand binding processes can be accompanied by protein and/or substrate protonation/deprotonation. Consequently, the ΔH observed depends not only on the intrinsic enthalpy but also the enthalpy related to an ionization event occurring on complex formation, as described in Eq. 6. Shikimate binding processes were thus evaluated in buffers with different enthalpies of ionization and the results are presented in **Fig. 6B** and **Table 2**. The results show a linear decrease to values of N_H+_ = −0.47±0.01 and a ΔH_int_ of −3.2 kcal mol^−1^ (Y-axis intercept corresponding to ΔH_ion_ = 0), indicating release of protons from the *Mt*SK:SKH binary complex to the bulk solvent. In other words, there are less protons associated with the binary complex than in the free interacting molecules, i.e. there is no sequestering of protons from bulk solution in the protein structure upon SKH binding to *Mt*SK. To try to address the possible source of proton released into solution, the p*K*_a_ values of amino acid side chains in the catalytic site were analyzed based on structural information [23]. Employing the p*K*_a_ values of 3.9, 4.47 and 12.5 for, respectively, Asp (β-COOH), SKH (1-COOH) and Arg (δ-guanidino) amino acid residues in solution, the change in the protonated fraction of a reactant species at a fixed pH value can be inferred by the Henderson-Hasselbalch equation (**Eq. 7**). This analysis permits to predict that there is a proportion of 10^−4.9^ of protonated Arg side chain (either Arg58 or Arg136) at pH 7.6. SKH and Asp34 at pH 7.6 show a proportion of, respectively, 10^+3.13^ and 10^+3.7^ of deprotonated (conjugate bases) in relation to protonated forms. Hence, the δ-guanidinium groups of Arg side chains located in the catalytic site (Arg58 and Arg136) are likely the proton donors to bulk solvent, which could account for the release of protons from *Mt*SK:SKH binary complex into solution. The latter will convert the salt bridge between the Arg side chain and the carboxyl group of SKH into hydrogen bonds between the reacting species. In agreement with this proposal, these interactions have been shown for SKH in complex with *Mt*SK [19], [21], [22], [23], [43,]. Interestingly, the *K*_D_ value for SKH in imidazole buffer (Table 2) appears to be larger as compared to *K*_D_ values in Hepes and Pipes. To assess whether or not imidazole has any inhibitory effect on *Mt*SK activity, measurements of enzyme velocity in the presence of this chemical compound were carried out. No enzyme inhibition could be observed in assay mixture containing 50 mM of imidazole (9.5 U mL^−1^) as compared to assay mixture in the absence of imidazole (9.2 U mL^−1^). Interestingly, SKH has been shown to bind to *Mt*SK with half occupancy [23]. It is thus tempting to suggest that the value of −0.47 for N_H+_ may reflect this structural feature of *Mt*SK:SKH binary complex formation.

**Figure 7:**
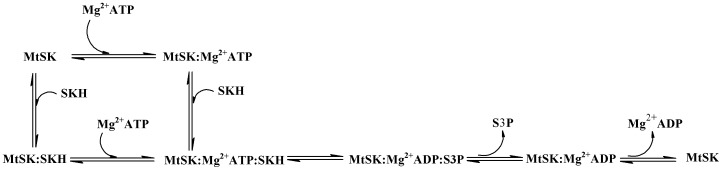
Enzyme mechanism of *Mt*SK.

It has been shown for *aroL*-encoded SK from *Erwinia chrysanthemi* (*Ec*SK) that the *K*_M_ for ATP (620 µM) is approximately four times lower than its *K*_D_ value (2.6 mM) [54]. These results prompted the proposal that the conformational changes in *Ec*SK associated with binding of the first substrate leads to an increase in the affinity for the second substrate. However, based on the results for Mg^2+^ATP, it does not appear to hold for *Mt*SK since the *K*_M_ value (112 µM) is in the same concentration range of *K*_D_ determined from ITC measurements (196 µM). It has been put forward that the *K*_M_ value for a substrate in rapid-equilibrium random-order mechanisms is equal to the equilibrium dissociation constant for dissociation of the substrate from the ternary complex [55]. The *K*_M_ value for SKH (**Table 1**, 650 µM) is approximately 3.6-fold larger than its *K*_D_ value (**Table 2**, 181 µM). These results suggest a positive free energy coupling (ΔG_coop_ = 3.2 kJ mol^−1^) for SKH binding to *Mt*SK:Mg^2+^ATP. There thus appears to be a negative cooperativity (ΔG_coop_ > 0) [56] in energy coupling of Mg^2+^ATP binding to *Mt*SK on SKH binding to the binary complex and ensuing ternary complex formation. This finding is somewhat puzzling because one would expect that Mg^2+^ATP binding to *Mt*SK should result in increased affinity for SKH. However, this proposal should be taken with caution as there may be additional step(s) that should be added to the simple mechanism depicted in **Fig. 7**. The energy conservation law dictates that the product of *K*_D_ and *K*_M_ with SKH binding first should give a value equal to (or approximately the same) the product of *K*_D_ and *K*_M_ with Mg^2+^ATP binding first. There is, however, a 6-fold difference for the product of dissociation constants between the routes from the apo enzyme to ternary complex formation. This discrepancy could tentatively be ascribed to either an additional step that could not be detected by the experimental approaches employed or to result from having treated the *K*_M_ values as “true” (microscopic) dissociation constants. Interestingly, no direct interaction between SKH and the LID domain of *Mt*SK was seen when SKH binds as the second ligand to the nucleotide-bound enzyme [23]. It is tempting to suggest that these reduced interactions may account for the larger *K*_M_ value as compared to *K*_D_ for SKH.

## Summary

Steady-state kinetics, fluorescence spectroscopy and isothermal titration calorimetry data showed that the enzyme mechanism of monomeric *Mt*SK is rapid-equilibrium random order of substrate addition, and ordered product release with S3P being released first followed by ADP dissociation from the binary complex to regenerate free enzyme (**Fig. 7**). The thermodynamic signatures of non-covalent interactions obtained from ITC data upon substrate(s)/product(s) binding to *Mt*SK demonstrated conformational changes following nucleotide binding and release of “bound” water molecules from SKH and/or *Mt*SK active site to bulk solvent. Results of the dependence of enthalpy on both temperature and buffer ionization upon *Mt*SK:SKH binary complex formation suggested a large hydrophobic contribution to substrate binding and indicated an important role of Arg58 and Arg136 side chains in SKH binding. Interestingly, although the genome sequencing of *M. tuberculosis* H37Rv strain has identified *Mt*SK as an *aroK*-encoded enzyme (SK I) [42], which has a large value for the Michaelis-Menten constant of SKH, the measurements of true steady-state kinetic parameters presented here and elsewhere [43] show that *Mt*SK would more appropriately be described as an *aroL*-encoded type II enzyme (SK II). Incidentally, it has recently been pointed out that understanding the mode of action of an enzyme can be used to inform functional annotation of newly determined sequences and structures, to select appropriate enzyme scaffolds for engineering new functions, and to refine definitions in the current EC classifications [57].

The currently available repertoire of antimycobacterial agents reveals only a handful of comprehensively validated targets, namely RNA polymerase, DNA gyrase, NADH-dependent enoyl-ACP reductase and ATP synthase [58]. The complete genome sequencing of *M. tuberculosis* H37Rv strain has accelerated the study and validation of molecular targets aiming at the rational design of anti-TB drugs [42]. The target-based rational design of new agents with anti-TB activity includes functional and structural efforts. Accordingly, mechanistic analysis should be included in enzyme-targeted drug programs aiming at the rational design of potent enzyme inhibitors. Moreover, ITC has been used as an important technique for the direct determination of thermodynamic and kinetic parameters of enzymatic reactions [59]. As mentioned above, the recognition of the limitations of high-throughput screening approaches in the discovery of candidate drugs has renewed interest in ITC data in the rational design of chemotherapeutic agents [6]. Moreover, understanding the mode of action of *Mt*SK will inform us on how to better design inhibitors targeting this enzyme with potential therapeutic application in TB chemotherapy. It is thus hoped that the results here described may be useful to the rational design of anti-TB agents and that they may contribute to our understanding of the biology of *M. tuberculosis*.
